# Genomic markers of panitumumab resistance including ERBB2/HER2 in a phase II study of KRAS wild-type (wt) metastatic colorectal cancer (mCRC)

**DOI:** 10.18632/oncotarget.8006

**Published:** 2016-03-09

**Authors:** Garrett S. Barry, Maggie C. Cheang, Hector Li Chang, Hagen F. Kennecke

**Affiliations:** ^1^ Department of Pathology and Laboratory Medicine, Faculty of Medicine, University of British Columbia, Wesbrook Mall, Vancouver, BC V6T 2B5, Canada; ^2^ The Institute of Cancer Research, Sutton, London, Surrey SM2 5NG, United Kingdom; ^3^ Medical Oncology, BC Cancer Agency, Vancouver, BC V5Z 4E6, Canada

**Keywords:** mCRC, nanostring, ERBB2, HER2, EGFR inhibitor resistance

## Abstract

A prospective study was conducted to identify biomarkers associated with resistance to panitumumab monotherapy in patients with metastatic colorectal cancer (mCRC). Patients with previously treated, codon 12/13 *KRAS* wt, mCRC were prospectively administered panitumumab 6 mg/kg IV q2weeks. Of 34 panitumumab-treated patients, 11 (32%) had progressive disease at 8 weeks and were classified as non-responders.

A Nanostring nCounter-based assay identified a 5-gene expression signature (*ERBB2*, *MLPH, IRX3*, *MYRF*, and *KLK6*) associated with panitumumab resistance (*P* = 0.001). Immunohistochemistry and *in situ* hybridization determined that the HER2 (*ERBB2*) protein was overexpressed in 4/11 non-responding and 0/21 responding cases (*P* = 0.035). Two non-responding tumors had *ERBB2* gene amplification only, and one demonstrated both *ERBB2* amplification and mutation. A non-codon 12/13 *KRAS* mutation occurred in one panitumumab-resistant patient and was mutually exclusive with *ERBB2*/HER2 abnormalities.

This study identifies a 5-gene signature associated with non-response to single agent panitumumab, including a subgroup of non-responders with evidence of aberrant *ERBB2/HER2* signaling. *KRAS* wt tumors resistant to EGFRi may be identified by gene signature analysis, and the HER2 pathway plays an important role in resistance to therapy.

## INTRODUCTION

Colorectal cancer (CRC) has high rates of metastasis and recurrence [1, 2]. Therapy for advanced disease includes epidermal growth factor receptor (EGFR)-inhibitors, including cetuximab and panitumumab, which have only shown effectiveness in *RAS* non-mutated tumors, though demonstrating a marked range of clinical response rates [3–5]. This heterogeneous response rate may be due to substantial intrinsic molecular differences among these tumors, including, but not limited to, mitogen-activated protein–kinase (MAPK/ERK) and phosphoinositide 3-kinase (PI3K/Akt) pathways, and anti-apoptosis [6–8]. Phase III trials of patients with metastatic *KRAS* unselected mCRC treated with single agent EGFR inhibitors (EGFRi) reported a high rate of resistance to therapy, with 70% of patients having progressive disease as best response [3]. Excluding patients with *KRAS* mutated tumors and other recently described *RAS* mutants [9, 10], reduces this proportion to approximately 30% of patients without response to EGFRi therapy. Determining which patients are unlikely to respond to EGFRi therapy will spare the patient from treatment-related toxicity and allow them to be directed to other therapeutic options or clinical trials, thus gaining time.

Inter-tumoral heterogeneity in CRC has been demonstrated in whole genome expression array studies, with the identification of six and three biological subtypes in two large, recent cohorts, respectively [7, 11]. Furthermore, gene expression-based drug sensitivity predictors have been shown to be the superior to other genomic, epigenomic, and proteomic drug sensitivity predictors across human breast cancer cell lines [12]. Gene expression signatures already have an established role in risk stratification and treatment selection in some settings, most notably in early stage breast and colon cancers [13–15].

In this phase II biomarker study, we aimed to identify molecular markers in *KRAS* wt mCRC that would identify patients resistant to EGFRi therapy. We determined the presence of molecular determinants of primary resistance to EGFRi therapy, defined as disease progression within 8 weeks of commencing single agent therapy with panitumumab. Gene expression profiling was conducted with a knowledge-based selection of candidate genes and targeted next-generation sequencing was performed. *ERBB2*/HER2 copy number status and protein expression quantification was conducted due to the identification of this gene in the response signature.

## RESULTS

**Figure 1:**
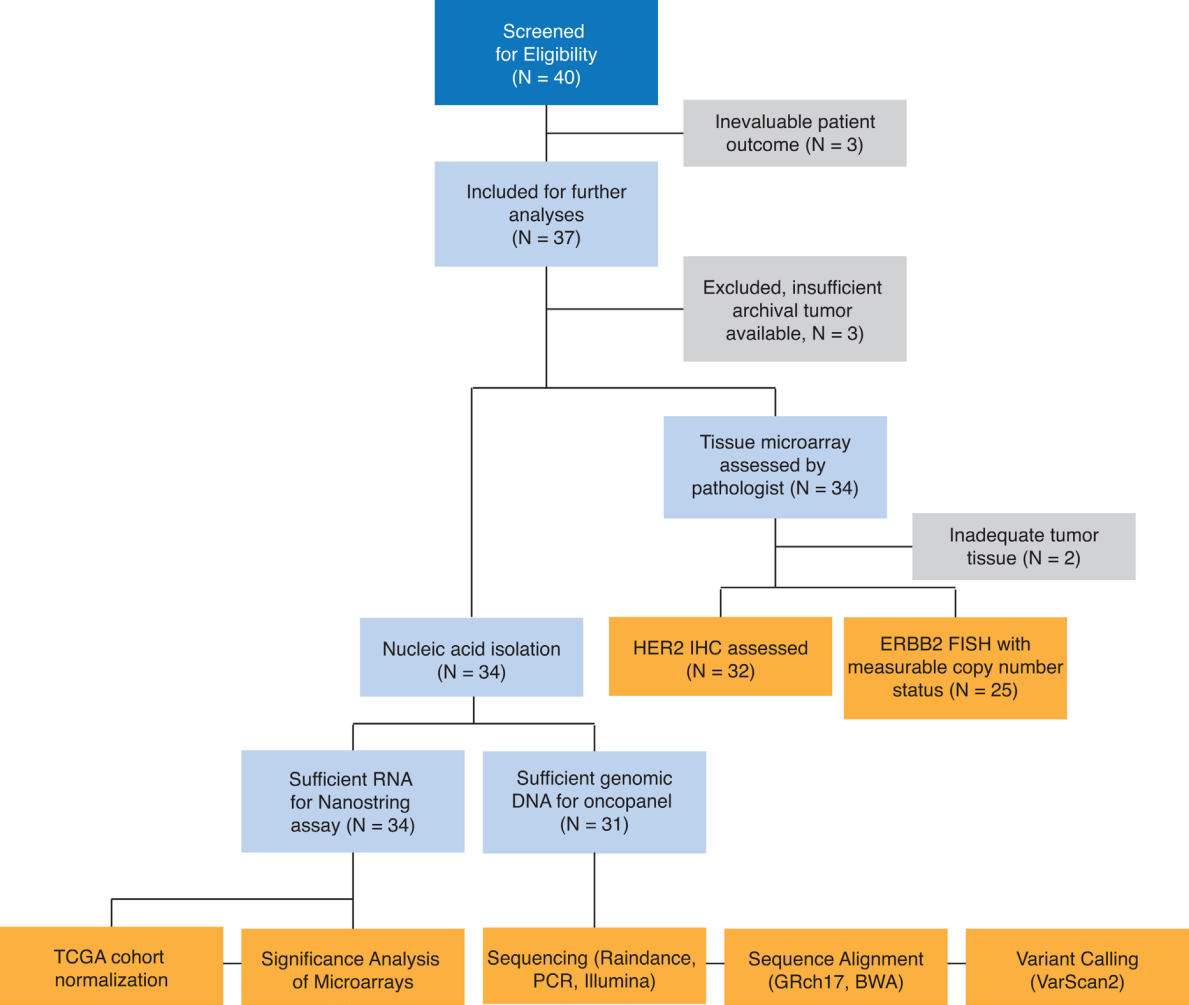
Design and description of sample collection, experimental steps, and analytical workflow

Of the 37 evaluable patients, one demonstrated a false negative *KRAS* codon 12 mutation and an additional two patients did not have sufficient primary tumor nucleic acid isolation; these patients were removed from subsequent analyses (Figure 1). Of 34 remaining evaluable cases, the median age was 64.5 years. All patients received prior chemotherapy including 59% who received 5-Fluorouracil, irinotecan and oxaliplatin and 53% received prior bevacizumab. Response to therapy and median number cycles of panitumumab are summarized in Table 1. Eleven of the remaining 34 patients developed progressive disease (PD) (median # of cycles = 4) at the time of the initial imaging for response at the 8-week time point and were classified as non-responders. Of the remaining 23 patients, nine had stable disease (SD) and eight had a partial response (PR) (median # cycles = 12). Six patients demonstrated a best response of two-year prolonged stable disease (median # cycles = 22). For the purposes of the biomarker analysis, patients were dichotomized into response groups and defined as non-responders if they had PD (*n* = 11) and responders if they had SD, PR, or PSD.

**Table 1:** Characteristics and response to therapy of 34 patients with previously treated *KRAS* wt mCRC

Total	34 (%)
**Median age**	64.5
**Prior Therapy**	
5-Fluorouracil (5-FU)/Capecitabine (C) only	10 (29)
5-FU/C and Irinotecan	1 (3)
5-FU/C and Oxaliplatin	3 (9)
5-FU/C, Irinotecan and Oxaliplatin	20 (59)
**Prior Bevacizumab**	
Yes	18 (53)
No	16 (47)
**Response to therapy**	
Progressive Disease	11 (32)
Stable Disease	9 (26)
Prolonged Stable Disease	6 (18)
Partial Response	8 (24)
Complete Response	0 (0)
**Median number of cycles of q2weekly therapy**	
Progressive Disease	4
Stable Disease	8
Partial Response/Prolonged Stable Disease	16

### Gene expression analysis identified 5 genes significantly associated with non-response to panitumumab

**Figure 2:**
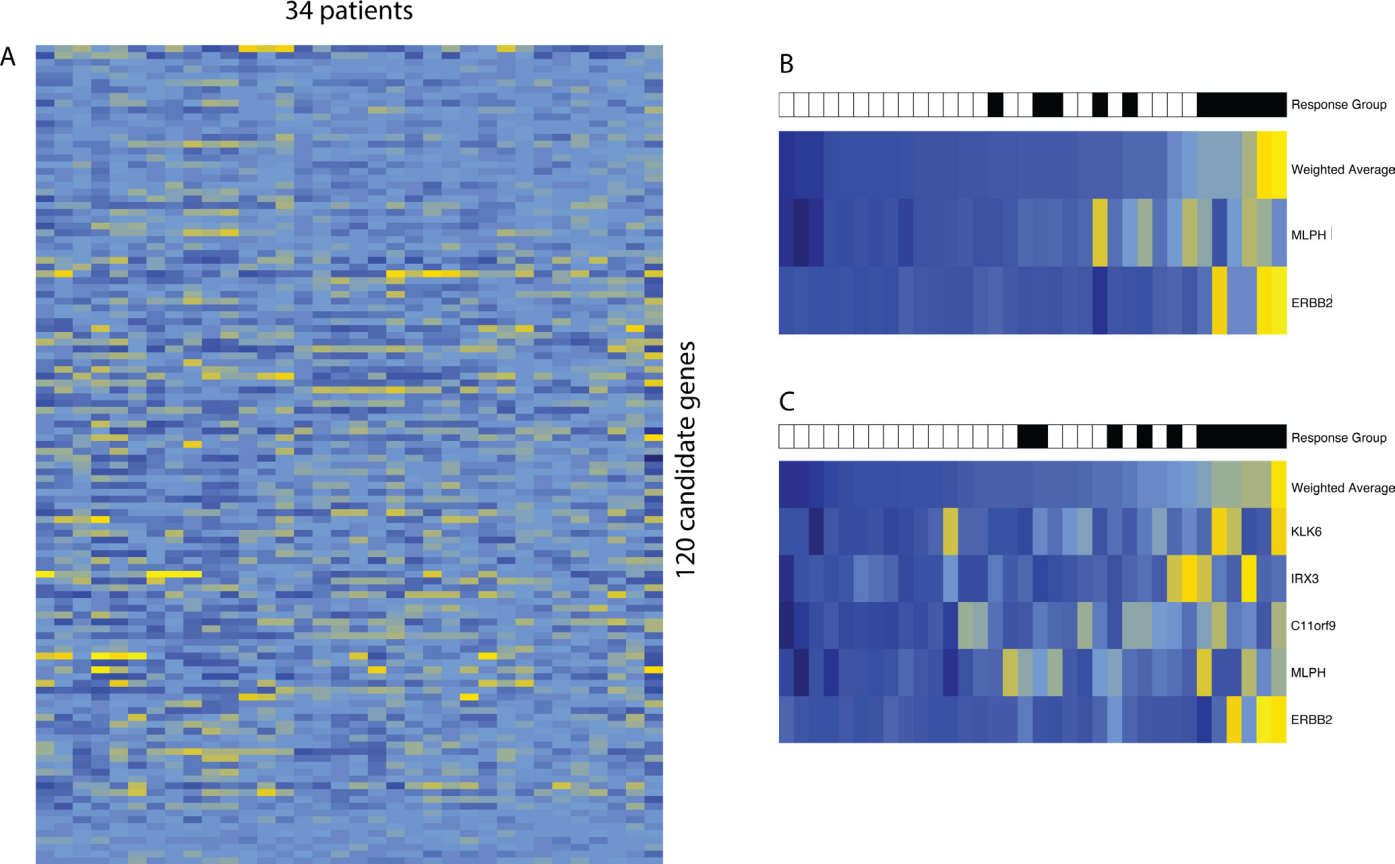
Gene expression results (**A**) Unsupervised hierarchical clustering of 34 cases of the *KRAS* wt mCRC cohort (BCCA) by 120 selected genes. Metastatic cases substituted primary tumor cases when available. (**B**) SAM analysis of selected 120 genes ranked two top genes with 0% FDR, *ERBB2* and *MLPH*. Cases were ordered by weighted average score of these two genes from low to high. Response to panitumumab was added and shows clustering with higher weighted average score (*P* = 0.05, CI 95% = 0.0015–1.98). (**C**) With an FDR cutoff of 15%, the addition of three genes, *IRX3*, *MYRF*, and *KLK6*, improved clustering of weighted average score with panitumumab non-responders (*P* = 0.001, CI 95% = 0.597–2.16). Non-responders represented patients with progressive disease (black), while responders encompassed stable disease, partial response, and prolonged stable disease (white).

We identified five highly ranked genes with a positive correlation with panitumumab resistance below an FDR of 15% by Significance Analysis of Microarrays (SAM); *ERBB2*, *MLPH*, *IRX3*, *MYRF*, and *KLK6* (Figure 2). Two of these genes, *ERBB2* and *MLPH*, had a FDR of 0%. These two genes alone were borderline significantly associated with non-response to panitumumab (*P* = 0.05, CI 95% = 0.0015–1.98), but the five-gene signature demonstrated a more significant association (*P* = 0.001, CI 95% = 0.597–2.16). Recognizing the weakness of a small cohort size, we performed normalization with the 220 cases of *KRAS* wt mCRC from the TCGA cohort. Ninety-five of the 120 genes had matched expression between BCCA and TCGA cohorts with over 30% of cases above background expression after normalization. Performing identical subsequent analyses on the TCGA-normalized data identified four highly ranked genes with an FDR below 10%, *ERBB2, MLPH, IRX3,* and *MYRF* (Supplementary Figure 2), and demonstrated very significant association with panitumumab response (*P* = 2.86 × 10^−8^, CI 95% = 1.02–1.89). A comparison of all three analyses can be found in Supplementary Figure 3. While re-performing the analysis on a smaller set of genes may have inflated the level of significance, taken together, this analysis bolstered the significant genes found in the SAM analysis prior to this normalization method. We noted that *EGFR* expression was not significantly associated with panitumumab response in any of these analyses, despite being the direct target of panitumumab.

### Mutations in *RAS*, BRAF, and ERBB2

Thirty-one out of 34 evaluable primary tumor cases included in the study had sufficient genomic DNA and matching blood DNA to be sequenced by the Illumina-based Oncopanel. Ten out of 11 matching metastatic tumor samples also passed these criteria and were sequenced.

**Figure 3:**
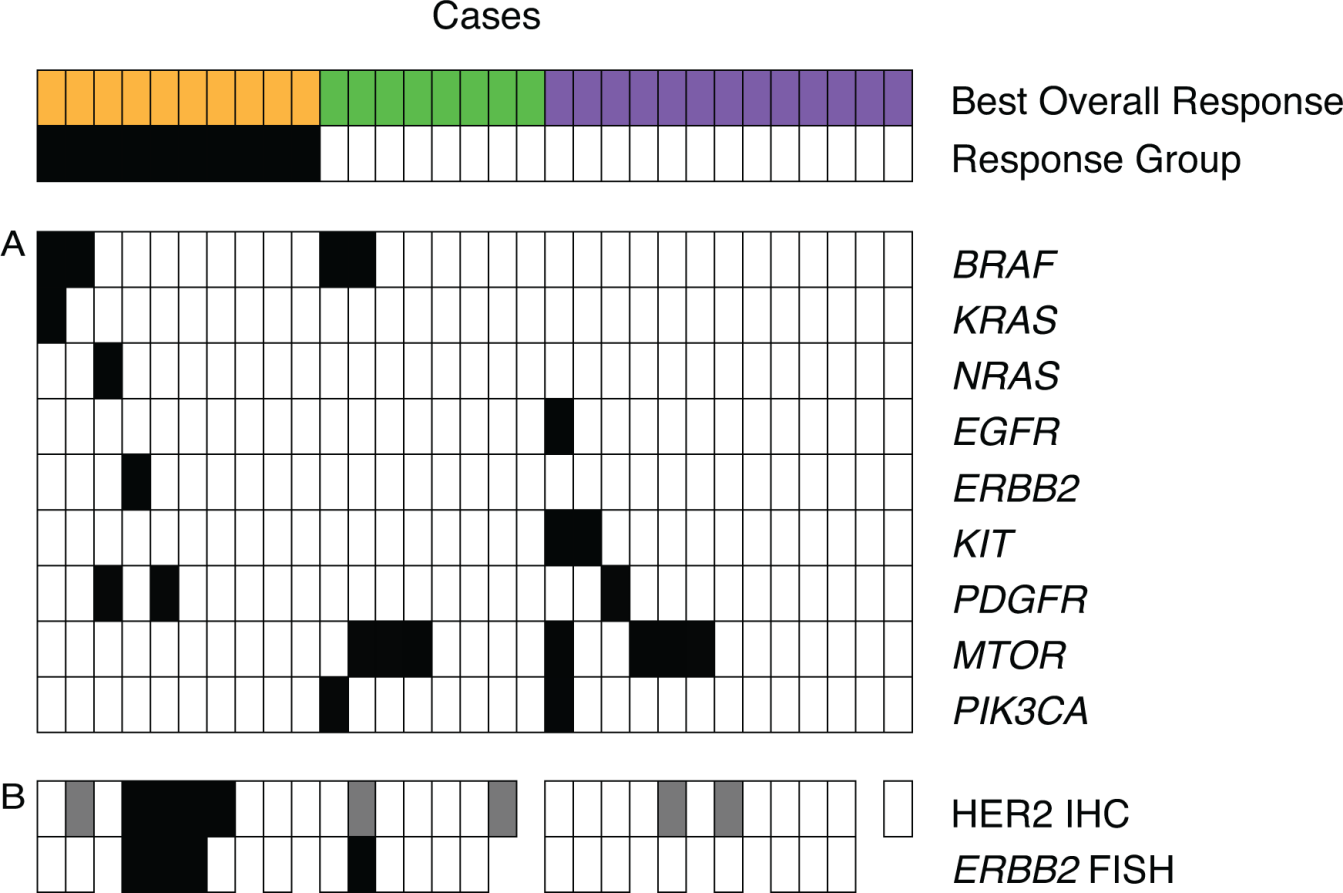
Mechanisms of panitumumab resistance in KRAS wt mCRC primary tumors may be explained by escaping wt EGFR dependence (**A**) Oncopanel sequencing of *BRAF*, *KRAS*, *NRAS*, *EGFR*, *ERBB2*, *KIT*, *PDGFR*, *MTOR*, and *PIK3CA* in 31 patients with best overall response of progressive disease (orange), stable disease (green), and partial response or prolonged stable disease (purple). Gene mutated = black, wild-type = white. (**B**) IHC demonstrated the HER2 protein expression (Scoring: black = 3+, grey = 1–2, white = 0) and FISH showed the ERBB2 gene copy number (black: > 2.1 copies; average of 20 cells/sample). Response groups: non-responders = black, responders = white.

Of the 31 primary tumor samples, one *KRAS* mutation was found in a non-responding patient (Figure 3). This G to A transition mutation produced an A146T amino acid change. A mutation in *BRAF* (K601E) was also found in this *KRAS* A146T mutant patient. This A146T mutation was also identified in a second patient with PD that was ultimately excluded from the study on the basis of unavailable RNA for gene expression analysis. One *NRAS* mutation at Q61L was also identified in a patient with progressive disease. No *HRAS* mutations were identified.

**Table 2:** List of gene mutations in 31 primary tumors by best overall response to panitumumab treatment identified within the Illumina-based sequencing oncopanel

Gene	Progressive Disease (PD)	Stable Disease (SD)	Partial Response (PR)/Prolonged stable disease (PSD)
	*N* = 10 (%)	*N* = 8 (%)	*N* = 13 (%)
*BRAF*	2 (20)	1 (13)	0
*EGFR*	0	0	1 (8)
*KRAS*	2 (20)	0	0
*HRAS*	0	0	0
*NRAS*	1 (10)	0	0
*AKT1*	0	0	0
*ALK*	0	0	0
*ERBB2*	1 (10)	0	0
*IDH1*	0	0	0
*IDH2*	0	0	0
*KIT*	0	0	2 (15)
*MAPK1*	0	0	0
*MAP2K1*	0	0	0
*MTOR*	0	3 (38)	4 (31)
*PDGFRA*	2 (20)	0	1 (8)
*PIK3CA*	0	1 (13)	1 (8)
*PTEN*	0	0	0
*STAT1*	1 (10)	1 (13)	1 (8)
*STAT3*	1 (10)	0	2 (15)
*TP53*	9 (90)	7 (88)	11 (85)

A total of four *BRAF* mutations were identified in the 31 patients, two in patients with PD and two in patients with SD. One of the two patients with PD had the V600E mutation and the other with a K601E mutation. Of patients with SD, one had a K601E mutation and the other had an L597R mutation. One patient classified as having a partial response had a frame shift mutation in EGFR itself, likely abrogating EGFR pathway activation.

There was one patient with a mutation in *ERBB2* classified as a non-responder. This was a homozygous V747L mutation that occurred in the tyrosine kinase domain of the protein; however, we are unaware of any study reporting the functional significance of this mutation at the time of this study.

Three patients had *PDGFRA* mutations, of which two were EGFRi non-responders. One of these *PDGFRA* mutations occurred in a resistant patient with a coexisting *NRAS* mutation. *KIT* mutations were found in two patients within the responder group. Infrequent mutations in MTOR and PIK3CA were also found in both responders and non-responders.

### Immunohistochemistry and *FISH* demonstrate over-activity of the HER2 pathway in a subset of RAS-BRAF wt panitumumab non-responders

Of the 35 assessable TMA cases, two did not undergo the gene expression assay and one had *KRAS* mutated codon 12, so were excluded as described earlier. Twenty-six out of 32 cases were negative (score = 0), 5/32 cases were low (score = 1), and 4/32 cases were high (score = 3+) for HER2 protein expression (Figure 3). The level of HER2 protein expression was significantly associated with resistance to panitumumab (Fisher's exact test, *P* = 0.035).

FISH was also performed on cases of the TMA with adequate tumor tissue and *KRAS* wt as for IHC, removing the same 3 cases from a total of 37. An additional 9 cases did not have adequate FISH results for assessment, leaving 25 cases with measurable *ERBB2* copy number. *ERBB2* amplification was correlated significantly with panitumumab resistance (*P* = 0.009, CI 95% 0.418–2.68). Four cases were found to have amplification of *ERBB2* with HER2/CEP ratios of > 2.1 (Figure 3). Three of these amplified cases demonstrated PD after panitumumab and had average copy numbers exceeding 8 copies of *ERBB2*. The fourth amplified case had HER2/CEP ratio of 2.2 and demonstrated stable disease.

## DISCUSSION

The objective of this prospective, exploratory biomarker study was to identify biomarkers associated with resistance to panitumumab therapy in *KRAS* wt mCRC on archival paraffin embedded tissue. Eleven out of 34 patients (32%) with *KRAS* wt (codons 12/13) mCRC did not respond to single agent panitumumab. A 5-gene signature was developed including *ERBB2* and *MLPH* on the Nanostring^®^ nCounter platform, and strongly associated with non-response to therapy. HER2 protein amplification was present in 13.8% (4/29) of all-*RAS* wt cases and was significantly associated with non-response to therapy.

A recently described 180-gene predictor assay was found to significantly classify EGFR-inhibitor-treated lung and colon adenocarcinoma disease types into high and low response groups [16]. The authors found 26 genes from the 180-gene set were adequate to significantly classify high and low cetuximab responders in *KRAS* wt mCRC. These genes were represented in the PI3K/AKT and MAPK pathways downstream of EGFR. A separate phase I dose-escalation cetuximab trial in a *KRAS* wt mCRC cohort reported a non-overlapping set of 6 genes associated with response to EGFR inhibition, though the biological significance of these genes is currently unknown [13]. None of these 6 genes were represented in the expression signature of the current study.

A subset of poor-prognosis *BRAF*-mutant-like (*BRAF*m) patients, defined as having tumors with gene expression profiles similar to known *BRAF*m tumors, were previously identified in a *KRAS* wt CRC cohort using a predictor based on differential expression of 32 gene pairs [17]. Remarkably, the *BRAF*m predictor identified 13% of *KRAS* wt CRC patients with a much poorer prognosis (*P* = 0.022), capturing a significant number of non-*BRAF* mutant patients with a *BRAF*m expression signature. We included these 64 genes in the present study, reasoning a *BRAF*-mutant-like phenotype may be associated with non-response to EGFR-directed therapies. Indeed, *MYRF, MLPH,* and *IRX3* expression - genes implicated in the *BRAF*-mutant-like phenotype - were associated with non-responders. Overexpression of these three genes was most significantly associated with panitumumab non-response aside from *ERBB2,* and thus may represent a relevant molecular pattern of panitumumab resistance by aberration and/or bypass of the EGFR signaling pathway for cancer cell survival. *IRX3* may be implicated in colorectal tumorigenesis by reducing tumor cell sensitivity to the Dpp/TGFβ pathway, granting tumor cells a growth advantage [18]. *MLPH* and *MYRF* have not been specifically described as linked to EGFRi resistance in CRC tumors, so this is a novel finding of this study.

The presently described outcomes are comparable with other studies. Peeters *et al*. reported 16% of patients with *KRAS* wt (codons 12/13/61) tumours showed no objective response to panitumumab monotherapy [9]. Among patients with *KRAS* wt (codons 12/13/61), nine and 13 out of 138 patients showed mutations in *NRAS* and *BRAF*, respectively, all of which showed no objective response to panitumumab. Patients with mutant *TP53* and *PIK3CA* showed response rates of 17 and 20%, respectively. Doulliard *et al*. found that additional mutations in *KRAS*, *NRAS*, and *BRAF* in patients with *KRAS* wt codons 12/13 conferred an inferior progression free survival and overall survival for those treated with panitumumab-FOLFOX4 therapy [10]. Seventeen percent of *KRAS* wt codons 12/13 patients had these additional *RAS* mutations. Of note, one patient in our study had an A146T mutation in *KRAS*, unreported by routine clinical hotspot mutation assays. Previous evidence suggests that *KRAS* A146T mutations may have oncogenic potential in other cancer types [18–20]. Of note, the *KRAS* mutant patient also bore a K601E mutation in *BRAF*, an exceedingly rare occurrence. We suspect that the two mutations may have contributed to a lack of response to panitumumab in this patient [21].

Interestingly, four *BRAF* mutations were identified in the cohort, two in patients with resistant tumors and two in patients with stable disease. *BRAF* mutation is a powerful prognostic marker in mCRC conferring a significantly shorter overall survival regardless of type of systemic therapy [22, 23]. Despite some evidence [22], mutations in *BRAF* have not been consistently established as a predictive marker for EGFRi resistance, and current treatment recommendations do not exclude these patients from EGFRi therapy. However, in a large randomized clinical trial of irinotecan plus panitumumab versus irinotecan alone, the latter was favored in cases harboring *BRAF* mutations [24]. The variability of *BRAF* as a marker of EGFRi resistance is reflected in this study as *BRAF* mutations occurred in both 20% of resistant (PD) and 9.5% of responsive (SD/PR/PSD) tumors. This may highlight the value of a gene response signature to further distinguish resistant from responsive disease in *BRAF* mutant mCRC.

The significance of mutations found in *PDGFRA* and *KIT* are currently unclear. Activating *PDGFR* mutations have been implicated in a favorable tumor microenvironment and angiogenesis [25]. While possible that a relationship exists between *PDGFR* mutation and lack of response to EGFRi therapy, the low frequency of these mutations in the present cohort, as well as a confounding *NRAS* mutation, was not adequate to support this. However, the CORRECT clinical trial of multi-kinase (including *PDGFR*) inhibitor regorafenib in mCRC patients who previously failed panitumumab and/or cetuximab demonstrated a significant benefit both in progression-free and overall survival (HR = 0.5 and 0.71, respectively) [26]. This seems to suggest that a variety of tyrosine kinases, including PDGFR, may be involved in tumor resistance to agents targeting specific oncogenic targets, such as EGFRi. There have been no reports of *KIT* involvement in EGFRi responsiveness in the colorectal cancer literature, and the presence of these mutations in the responder group is of unknown significance.

We queried the TCGA data for alterations in the 5 top ranked genes from our gene expression analysis using the cBioPortal for Cancer Genomics from Memorial Sloan Kettering Cancer Center [27, 28]. Thirteen (6%) of the CRC cases from the TCGA database (*N* = 224) had gene alterations in *ERBB2*, 7 of which were gene amplifications (3.1%). All *ERRB2* amplified cases were mutually exclusive of alterations in *MYRF*, *MLPH*, *IRX3*, and *KLK6*. *KLK6* was altered in 4 cases (2%), *MYRF* was altered in 3 cases (1%), and *IRX3* was altered in 2 cases (1%). *MLPH* was altered in 1 case. The relatively low frequency of these aberrations may point to their specificity with regards to predictors for EGFRi resistance.

Of the genes we included in the expression analysis, *ERBB2* overexpression by FISH and IHC was strikingly and significantly associated with panitumumab non-response. Previous studies of have reported a rate of HER2 positivity of 2.0 to 4.3% as defined by HER2 protein expression of 3+ protein or FISH amplification > 2.1 [29–32] in colorectal tumors. One study reported an overall frequency of 2.3% in 2349 patients reported in the literature [33]. Concordance of HER2 positivity in paired samples was reported to be 96% [32].

Until recently it was thought that HER2 gene copy number was not predictive of EGFR-directed therapy response [34]. However, recent studies do suggest HER2 overexpression by gene amplification may indeed be related to poor outcome in *KRAS* wt mCRC patients treated with cetuximab or panitumumab [35]. In a study of 137 patient-derived xenograft (PDX) tumors, HER2 amplification was documented in a 13.6% of cases in patients with cetuximab resistant, *KRAS* wild-type tumors [33], a frequency similar to the one documented in the current study. Two representative *KRAS* wt, HER2 amplified, cetuximab resistant PDX tumors were treated with pertuzumab, an anti-HER2 monoclonal antibody preventing dimerization with HER partners including EGFR, in combination with either lapatinib, a dual EGFR/HER2 tyrosine kinase inhibitor, or cetuximab, and resulted in substantial and prolonged tumor regression, whereas pertuzumab alone had no effect. An earlier phase II study of herceptin, an anti-HER2 monoclonal antibody, plus cytotoxic therapy with irinotecan in patients with advanced HER2 positive (2+ or 3+ protein expression) mCRC, reported a response to therapy in 5 of 7 patients, but the study was closed due to poor accrual as only 8% of 138 screened patients were HER2 positive [29]. In the current study 4 of 29 (13.8%) patients with all-*RAS* wt tumors had HER2 overexpression by IHC and/or FISH. This incidence significantly increases the feasibility of a trial of a dual EGFR inhibitor combined with HER2 directed therapy in advanced HER2 positive mCRC. Indeed, a recent phase II study of combination trastuzumab plus lapatinib in *KRAS* wt and HER2 IHC 2/3+ and FISH + cancers met its primary efficacy endpoint and reported a 32% response rate in patients with advanced, treatment refractory cancer [36]. This lends strong support to *ERBB2* as relevant therapeutic biomarker in mCRC.

In conclusion, this study represents a primary effort to define a novel gene expression signature based on differentially expressed genes in *KRAS* wt mCRC not responsive to EGFRi, in particular, panitumumab monotherapy. A five-gene expression signature was found to be highly associated with panitumumab resistance in mCRC, with *ERBB2* and *MLPH* as the top differentially expressed genes. We intend to explore if this gene expression signature is unique to mCRC or has utility in other tumor sites as well. The secondary goal was to identify possible mechanisms of panitumumab resistance by targeted resequencing of implicated genes of the EGFR and HER2 signaling pathways and protein expression and gene amplification of HER2 and *ERBB2*, respectively. We detected molecular aberrations associated with EGFRi resistance in 6 out of 10 of non-responding patients. Further modeling and validation of the gene expression signature in a larger, independent cohort may allow refined prediction of resistant cases. A prospective screening method to identify patients least likely to respond to panitumumab monotherapy will spare patients from unnecessary exposure to EGFRi administration and toxicity and will help to redirect these patients to an alternative therapy or clinical trial earlier, enhancing their chances of a positive outcome.

## MATERIALS AND METHODS

### Patient eligibility and study design

A total of 40 patients with *KRAS* wt (codons 12 and 13) advanced mCRCs with measurable disease were prospectively enrolled at three BCCA cancer centers on an exploratory biomarker study. Patients were previously treated with 5-Fluorouracil and received, or were ineligible for, prior oxaliplatin/5-FU and irinotecan and were treated with panitumumab 6 mg/kg IV q2w until progression or toxicity. None of the patients enrolling on this study had ever been exposed to prior anti-EGFR therapy, either as a single agent or in combination with an irinotecan-based regimen. Computed Tomography (CT) was done at baseline and every 8 weeks to assess response. The primary endpoint was tumor response and patients were classified by standard RECIST criteria as Progressive Disease (PD), Stable Disease (SD), Prolonged Stable Disease ≥ 24 weeks (PSD) or Partial Response (PR). For the biomarker analysis, patients with PD were classified as non-responders and patients with SD, PSD24 or PR were classified as responders to panitumumab. The primary objective of the study was to determine rates of response and non-response by RECIST to single agent panitumumab therapy and the study was powered to detect biomarkers with a frequency of 20–50%. The study was approved by the institutional REB and was registered on clinicaltrials.gov study NCT00853931.

Sample collection, experimental steps, and analytical workflow are summarized in Figure 1. Of 40 *KRAS* wt mCRC patients treated with panitumumab monotherapy, 37 were evaluable for response to therapy. One patient with a *KRAS* mutation identified in codon 12 that was not identified by standard clinical testing was excluded from all analyses. Two samples that did not have sufficient RNA for the Nanostring assay requirements, nor did not pass expression data quality control determined by nSolver software (Nanostring), were also excluded, leaving a total of 34 patients included in the final analysis.

### Gene expression profiles

#### Gene selection and probe design

A 120-gene Nanostring nCounter codeset was designed from previously reported gene sets predictive of *BRAF* mutation status and cetuximab response in mCRC [13, 17, 37], and a hypoxia signature predictive of metastasis in a broad range of tumor types [38]. Additional genes of interest such as *RAD51*, *EGFR*, *ERBB2*, and *CRYAB* were also included [35, 39–42]. The complete gene list can be found in Supplementary Table 1.

### Nucleic acid isolation and expression profiling

Total ribonucleic acid (RNA) and genomic deoxyribonucleic acid (gDNA) was isolated from duplicate 1 mm diameter cores taken from FFPE tissue blocks with a Total Nucleic Acid Extraction kit (Qiagen). Qubit and Nanodrop-1000 spectrophotometry measured quantity and quality of RNA, respectively. Samples with a minimum quantity threshold of 12.5 ng/ul and a 260/280 quality ratio between 1.7 and 2.3 were included in the nCounter assay as previously described [43]. The nCounter assay was performed following recommended protocols using between 100 and 250 ng of total RNA.

### Data analysis

#### Gene expression data analysis

Data processing was performed in R Statistical Environment. The BCCA study cohort gene expression data was normalized to the same dimensional space as the wt cases from the 220-case TCGA cohort and sample-to-sample geomean normalized using eight 8 housekeeping genes: *ACTB*, *GUSB*, *PSMC4*, *RPLP0*, *PUM1*, *SF3A1*, *TFRC*, and *MRPL19*. Genes with expression above background in fewer than 30% of cases after normalization were removed. Principal component analysis (PCA) was used to visualize the dimensional space of the BCCA and TCGA cohort datasets.

Two-class unpaired Significance Analysis of Microarrays (SAM) was used to identify the top ranked genes associated with resistance to panitumumab in the BCCA cohort. For the cases where matched primary- and metastatic-derived tumor samples were both available, metastatic samples were included for SAM analysis in place of primary tumor samples. False Discovery Rate (FDR), or *q*-value, was used to identify the genes most significantly associated with panitumumab response. A T-statistic, called the score(d), was calculated by calculating a “d” statistic for each gene based on its linear regression with the outcome. “d” statistics are ordered and plotted against their expected order statistics after a large number of random permutations of the response data. The score(d) represented how far the “d” statistic is from the expected order statistic and was used to weight each corresponding gene within a signature equation. Weighted average expression of top ranked genes was calculated in each analyses and association with panitumumab response was performed by two-tailed unpaired Welch's *T*-test, which reports a 95% confidence interval for the difference in means of responders vs. non-responders.

Since the BCCA study cohort was composed entirely of *KRAS* wt tumors, we sought to identify if there was any expression bias of 120 selected gene expression profiles within a larger mixed *KRAS* wt and mutant CRC tumor cohort from The Cancer Genome Atlas (TCGA) database. Two hundred and twenty colon adenocarcinoma tumor samples with both global gene expression profiles and somatic mutational analysis were collected from TCGA. Gene expression and somatic mutation data from the TCGA cohort were previously measured with a custom Agilent 244K array and both SOLiD 4 and Illumina HiSeq platforms, respectively. Global gene expression profiles were abridged to only represent the 120 candidate genes.

Since the BCCA cohort size was relatively small and represented a biased population based in terms of *KRAS* wt status, we determined whether a gene expression bias was present between known *KRAS* mutant and wt cases. We assessed this by principal component analysis (Supplementary Figure 1). By normalizing the BCCA cohort dataset to the much larger TCGA cohort, the BCCA cohort dataset shifted to overlap with the TCGA cohort dataset correcting platform variation between datasets.

### DNA sequencing

Targeted resequencing of a panel of common oncogenes and tumor suppressor genes using an Illumina-based Oncopanel was performed. This sequencing panel included genes suspected to confer resistance to EGFR-directed therapy beyond *KRAS* codons 12 and 13 mutations, such as in other regions of the *RAS* homologs. A minimum of 250 ng of genomic DNA was submitted for Illumina-based Oncopanel targeted resequencing. The coding exonic sequence and at least 2bp of flanking intronic sequence of each target was determined from the source DNA material following RainDance-mediated primer-DNA droplet merging, PCR amplification and Illumina sequencing. Resulting sequences were aligned to the GRCh37 human genome reference using BWA (version 0.7.5a, mem algorithm). Variant calling was performed using VarScan2 (v2.3.6), with the minimum allele frequency threshold for reporting variants set to 0.1. cDNA nucleotide numbering begins at the A of the initiating codon (ATG) as per HGVS convention of the reference sequences (Supplementary Table 2).

### Immunohistochemistry and fluorescence *in situ* hybridization

As *ERBB2* gene expression showed the highest association in tumours that did not respond to panitumumab, immunohistochemistry (IHC) of HER2 protein and *ERBB2 in situ* hybridization (ISH) analysis was conducted. The antibody used for IHC was specific to HER2 protein (Ventana Medical Systems, Inc.). HER2-stained tissue microarray (TMA) slides were scored as 0 for negative, 1–2 for moderate expression, and 3+ as positive overexpression based on stain intensity according to the ASCO/CAP guidelines. Scores were defined as negative (0–2) or positive (3+) and analyzed by Fisher's exact test for significance.

For FISH analysis, TMA slides were stained with the Inform HER2 Dual ISH DNA Probe Cocktail Assay (Ventana Medical Systems, Tucson AZ) to determine HER2 gene amplification. Adequate staining was defined as the presence of both signals in at least 20 nuclei, performed on duplicate cores when possible. The numbers of HER2 and chromosome 17 signals were enumerated in 20 nuclei (only in nuclei containing both signals). HER2:Chromosome 17 ratios were calculated by dividing the number of HER2 signals by the number of chromosome 17 signals in each case; ratios of ≥ 2 were considered to have Her2 amplification. Significance of ISH results as a continuous variable to response was analyzed by Welch's two-tailed *T*-test, also reporting a 95% confidence interval for the difference in means between responders vs. non-responders.

Adjacent signals were only considered separate if the distance between the signals was greater or equal to one signal. Small clusters of HER2 signals where individual signal counting was impossible were counted as 6 signals, and large clusters were counted as 12 signals, as per the manufacturer's specifications.

## SUPPLEMENTARY MATERIALS FIGURES AND TABLES



## Abbreviations

BRAF*m*: BRAF-mutant-like
CT: Computed Tomography
CRC: colorectal cancer
EGFR: epidermal growth factor receptor
EGFRi: EGFR inhibitors
FDR: False Discovery Rate
FISH: fluorescence in situ hybridization
gDNA: genomic deoxyribonucleic acid
IHC: immunohistochemistry
ISH: in situ hybridization
MAPK/ERK: mitogen-activated protein–kinase
mCRC: metastatic colorectal cancer
PCA: Principal component analysis
PD: Progressive Disease
PI3K/Akt: phosphoinositide 3-kinase
PR: Partial Response
PSD: Prolonged Stable Disease
SAM: Significance Analysis of Microarrays
SD: Stable Disease
TCGA: The Cancer Genome Atlas
TMA: tissue microarray
*wt*: wild-type
