# Supplementary material for: Genomic markers of panitumumab resistance including ERBB2/HER2 in a phase II study of KRAS wild-type (wt) metastatic colorectal cancer (mCRC)
Source: Oncotarget. 2016 Mar 9;7(14):18953–64. doi: 10.18632/oncotarget.8006 (PMC4951343; doi:10.18632/oncotarget.8006)
Supplement: Supplementary file 1 [file oncotarget-07-18953-s001.pdf]

Genomic markers of panitumumab resistance including ERBB2/HER2 in a phase II study of KRAS wild-type (wt) metastatic colorectal cancer (mCRC)

Supplementary Materials

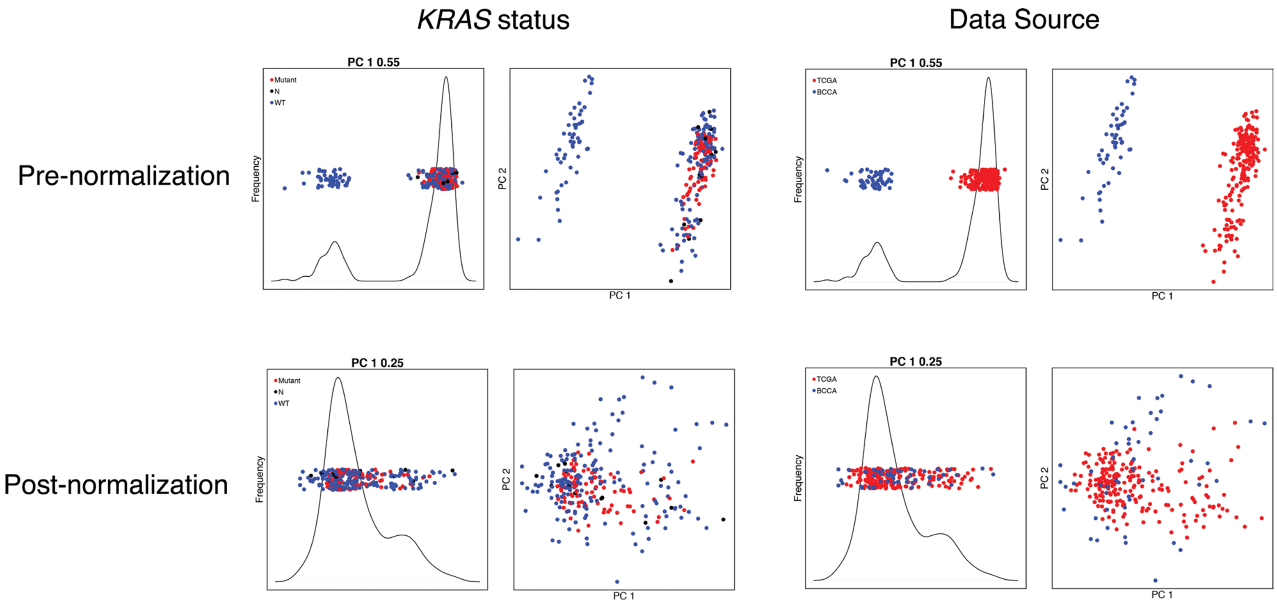

Supplementary Figure S1: Principal component analysis of the TCGA and BCCA cohorts before and after normalization. Prior to normalization, the two cohorts occupied non-overlapping dimensional space (top) but merge into the same dimensional space following normalization (bottom). *KRAS* mutant (red) and wt (blue) gene expression was normalized as to become overlapping (left). Data source of BCCA (blue) or TCGA (red) was also normalized to become overlapping (right).

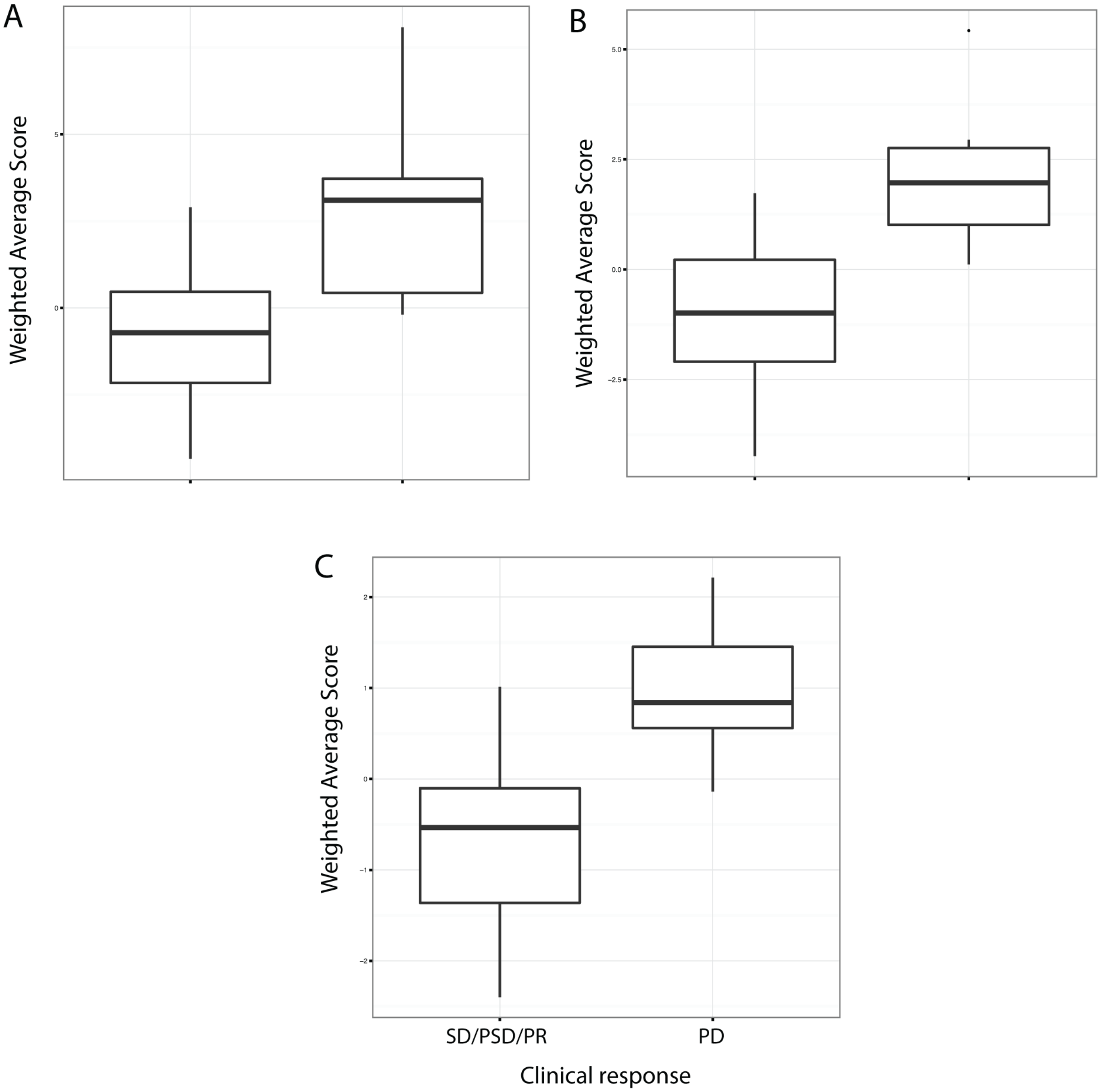

Supplementary Figure S2: SAM analysis of BCCA expression dataset after gene normalization with the TCGA expression dataset based on 95 genes present in both datasets and with at least 30% of cases demonstrating expression above background. Cases were ordered by weighted average score of four top ranked genes from low to high. Response to panitumumab shows significant clustering with higher weighted average scores (P = 2.86 × 10^{-8}, CI 95% = 1.02–1.89). Non-responders represented patients with progressive disease (black), while responders encompassed stable disease, partial response, and prolonged stable disease (white).

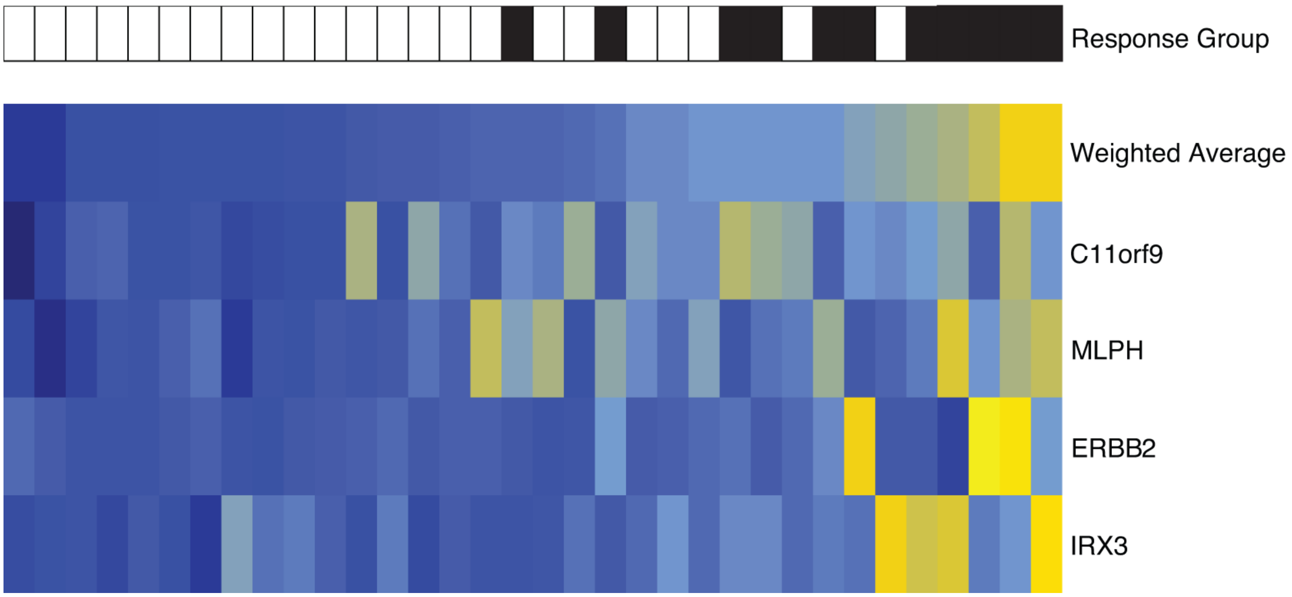

Supplementary Figure S3: Significance of multi-gene weighted score averages in predicting patients with resistance to panitumumab. (**A**) Two-gene weighted average scores of *ERBB2* and *MLPH* displayed borderline association with panitumumab responses of SD/PSD/PR versus PD (P = 0.05, CI 95% = 0.0015–1.98). (**B**) Association with response was improved in the five-gene expression signature measuring *ERRB2*, *MLPH*, *IRX3*, *c11orf9*, and *KLK6* (P = 0.001, CI 95% = 0.597–2.16). (**C**) Following normalization with the 220-case TCGA cohort, association of a four-gene expression signature with response was very significant ($P = 2.86 \times 10^{-8}$, CI 95% = 1.02–1.89). Box halves represent variance of second and third quartiles while lines represent variance of the first and fourth quartiles.

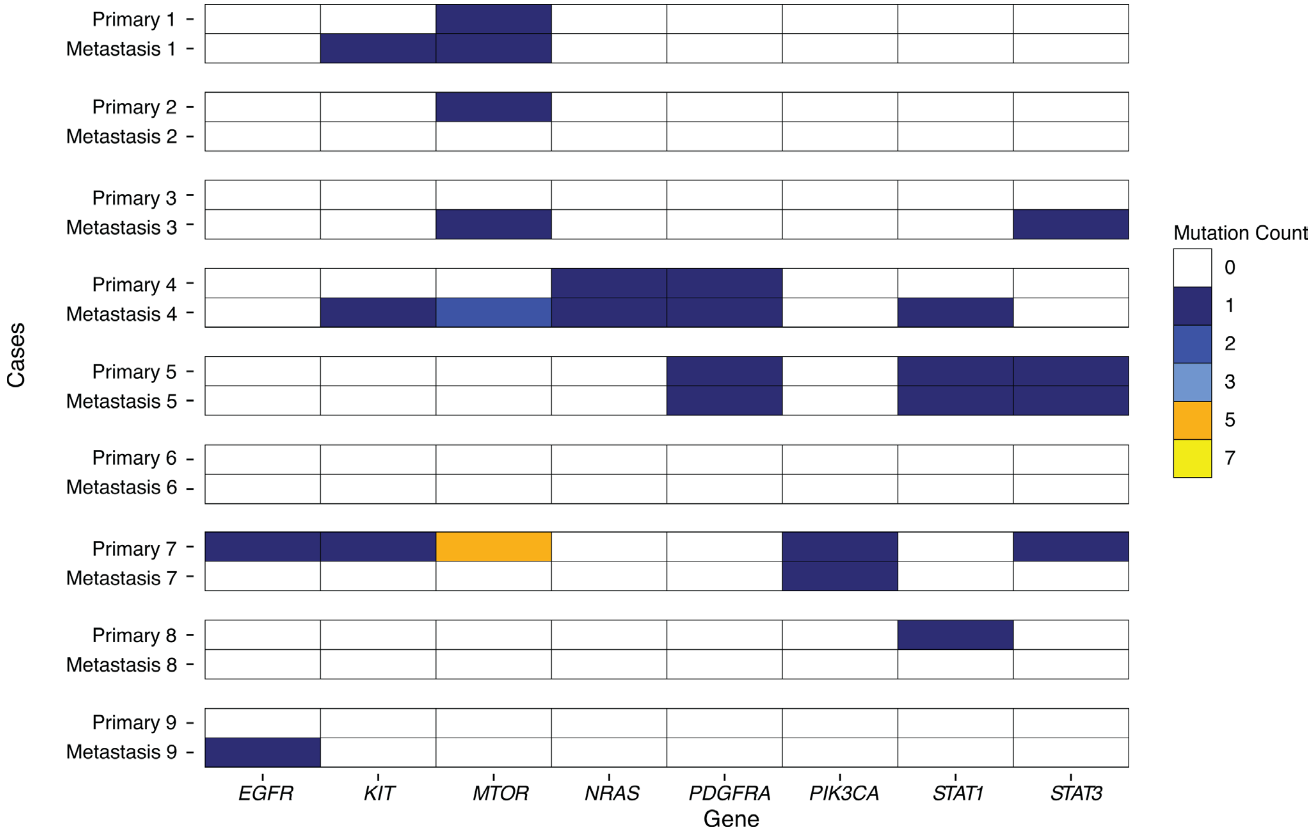

Supplementary Figure S4: Matched metastatic tumor samples displayed patterns consistent with a subclonal metastases or progressive evolution of tumor mutational status following treatment. Matched metastatic samples harbored mutations found in primary samples plus additional gene mutations. Metastatic cases 2, 7, and 8 lacked mutations found in the primary tumor samples. Pair 5 had no change between primary and metastatic tumor samples. Note: only genes with mutations in primary/metastatic pairs are shown.

Supplementary Table S1: List of 132 selected genes included in the nCounter codeset, compiled from gene sets implicated in cetuximab response and BRAF mutant-like expression patterns in other *KRAS* wt mCRC cohorts

C13orf18	OSBP2	RYK	MAPK13
CTSE	CFTR	HMOX1	RHOB
DDC	KLK10	GNB5	NUDT4
AQP5	PHYH	PIK3CA	ATP2C1
PPP1R14D	DUSP4	ELMO1	GNAS
REG4	PLCB4	GPSM2	CAMK2G
HSF5	HOXD3	PTK7	ITGA6
RSBN1L	ZNF141	TNFRSF1A	P2RY5
SATB2	C11orf9	ECOP	PRKD2
RASSF6	PPP1R14C	RAC1	CC2D1A
TNNC2	CD55	RGL2	TNFRSF1B
CRIP1	FLJ32063	TNFRSF10B	DNAJC8
GGH	TRNP1	PRKCI	ECSIT
PPPDE2	APCDD1	MAPK13	GOSR2
SPINK1	FSCN1	VEGFA	PPP1R9A
PLK2	ACOX1	RHOB	KLK6
PTPRO	KIAA0802	NUDT4	CRYAB
TM4SF4	C10orf99	ATP2C1	RAD51
ZSWIM1	PLLP	GNAS	EGFR
MLPH	MIR142	CAMK2G	ERBB2
RNF43	IRX3	ITGA6	RRAGD
RBM8A	ARID3A	P2RY5	FABP5
CELP	SLC25A37	PRKD2	UCHL1
SOX8	C20orf111	CC2D1A	GAL
CBFA2T2	PIK3AP1	TNFRSF1B	PLOD
PIWIL1	AMACR	DNAJC8	DDIT4
PTPRD	TPK1	PTK7	VEGFA
LOC388199	AIFM3	TNFRSF1A	ADM
CDX2	ZIC2	ECOP	ANGPTL4
S100A16	CTTNBP2	RAC1	NDRG1
TSPAN6	SERPINB5	RGL2	PNP
RBBP8	DDR1	TNFRSF10B	SLC16A3
VAV3	PRDX4	PRKCI	C14ORF58

Genes implicated in therapeutic resistance and anti-apoptosis have also been included [1−5].

Supplementary Table S2: Genes and reference sequences targeted by the oncopanel sequencing assay

Symbol	NCBI Reference Sequence	Locus Reference Genomic
HRAS	NM_005343.2	LRG_506
STAT1	NM_007315.3	LRG_111
STAT3	NM_139276.2	LRG_112
BRAF	NM_004333.4	LRG_299
EGFR	NM_005228.3	LRG_304
KIT	NM_000222.2	LRG_307
PDGFRA	NM_006206.4	LRG_309
PIK3CA	NM_006218.2	LRG_310
PTEN	NM_000314.4	LRG_311
TP53	NM_000546.5	LRG_321
KRAS	NM_033360.2	LRG_344
ALK	NM_004304.3	LRG_488
AKT1	NM_001014431.1	LRG_721
ERBB2	NM_001005862.1	LRG_724
MAP2K1	NM_002755.3	LRG_725
MTOR	NM_004958.3	LRG_734
NRAS	NM_002524.3	LRG_92
IDH1	NM_005896.2	LRG_610
IDH2	NM_002168.2	LRG_611
MAPK1	NM_002745.4	LRG_786

REFERENCES

1. Tennstedt P, Fresow R, Simon R, Marx A, Terracciano L, Petersen C, Sauter G, Dikomey E, Borgmann K. RAD51 overexpression is a negative prognostic marker for colorectal adenocarcinoma. Int J Cancer. 2013; 132:2118–2126.
2. Ruan Q, Han S, Jiang WG, Boulton ME, Chen ZJ, Law BK, Cai J. alphaB-crystallin, an effector of unfolded protein response, confers anti-VEGF resistance to breast cancer via maintenance of intracrine VEGF in endothelial cells. Mol Cancer Res. 2011; 9:1632–1643.
3. Tsang JY, Lai MW, Wong KH, Chan SK, Lam CC, Tsang AK, Yu AM, Tan PH, Tse GM. alphaB-crystallin is a useful marker for triple negative and basal breast cancers. Histopathology. 2012; 61:378–386.
4. Yonesaka K, Zejnullahu K, Okamoto I, Satoh T, Cappuzzo F, Souglakos J, Ercan D, Rogers A, Roncalli M, Takeda M, Fujisaka Y, Philips J, Shimizu T, et al. Activation of ERBB2 signaling causes resistance to the EGFR-directed therapeutic antibody cetuximab. Sci Transl Med. 2011; 3:3002442.
5. Martin V, Landi L, Molinari F, Fountzilas G, Geva R, Riva A, Saletti P, De Dosso S, Spitale A, Tejpar S, Kalogeras KT, Mazzucchelli L, Frattini M, et al. HER2 gene copy number status may influence clinical efficacy to anti-EGFR monoclonal antibodies in metastatic colorectal cancer patients. Br J Cancer. 2013; 108:668–675.
